# Need and Viability of Newborn Screening Programme in India: Report from a Pilot Study

**DOI:** 10.3390/ijns8020026

**Published:** 2022-03-29

**Authors:** Arya Raveendran, Teena Joseph Chacko, Priya Prabhu, Raghava Varma, Leslie Edward Lewis, Pragna Rao, Prajna P. Shetty, Yajna S. Phaneendra Mallimoggala, Asha Hedge, Dinesh M. Nayak, Sudeep Moorkoth, Sudheer Moorkoth

**Affiliations:** 1 Department of Pharmaceutical Quality Assurance, Manipal College of Pharmaceutical Sciences, Manipal Academy of Higher Education, Manipal 576104, India; arya.ravi@learner.manipal.edu (A.R.); teenajosephelavumkal@gmail.com (T.J.C.); priyaprabhu692@gmail.com (P.P.); raghavavarma0928@gmail.com (R.V.); 2 Department of Pediatrics, Kasturba Medical College, Manipal Academy of Higher Education, Manipal 576104, India; leslie.lewis@manipal.edu; 3 Department of Biochemistry, Kasturba Medical College, Manipal Academy of Higher Education, Manipal 576104, India; pragna.rao@manipal.edu (P.R.); prajnashetty85@gmail.com (P.P.S.); moggala.y@learner.manipal.edu (Y.S.P.M.); 4 Department of Pediatrics, Melaka Manipal Medical College, Manipal Academy of Higher Education, Manipal 576104, India; asha.hegde@manipal.edu (A.H.); dinesh.nayak@manipal.edu (D.M.N.); 5 Chesterfield Royal Hospital NHS Trust, Calow, Chesterfield S44 5BL, UK; s.moorkoth@nhs.net

**Keywords:** awareness, cost-effectiveness, India, incidence, newborn screening

## Abstract

India, a country with the second largest population in the world, does not have a national newborn screening programme as part of its health policy. With funding support from the Grand Challenges Canada, a pilot newborn screening programme was implemented for the Udupi district of South India to study the need and viability of a national programme in India. Six disorders were selected for the study based on the availability of funding and recommendation from pediatricians in the district. Here, we report the observed incidence during the study. A cost-effectiveness analysis of implementing newborn screening in India was performed. It is evident from our analysis that the financial loss for the nation due to these preventable diseases is much higher than the overall expenditure for screening, diagnosis, and treatment. This cost-effectiveness analysis justifies the need for a national newborn screening programme in India.

## 1. Introduction

The dried blood spot test introduced by Robert Guthrie in 1961 to screen phenylketonuria (PKU) initiated the concept of newborn screening (NBS) which became a national programme of the US in 1962 [1,2]. The benefits of newborn screening have formed a topic of discussion since then [3]. NBS helps in reducing preventable developmental delay, disability, morbidity and mortality during infancy and childhood. The outcome is a better quality of life for the affected child, with an increased cognitive, intellectual, and monetary contribution to society [4,5]. The decision to implement the NBS programme for a country largely follows the Wilson and Jungner criteria (World Health Organization, 1968). The most important criterion is that the disorders detected by a screening test should be treatable [6,7].

Since the bacterial inhibition assay used by Guthrie in 1961, many advancements have evolved in screening technology. The introduction of ELISA (enzyme-linked immunoassay), electrophoresis, high-performance liquid chromatography (HPLC) and liquid chromatography-mass spectrometry (LCMS) techniques helped in incorporating more disorders to the newborn screening panel. Currently, technology is available to screen more than 100 metabolic and genetic disorders. Recognizing the benefits of the newborn screening programme, many countries have implemented newborn screening programme as a part of their national health care policy. The disorders screened varies according to the local prevalence and based on the Wilson and Jungner criteria [8]. India has yet to implement a national newborn screening programme. The major obstacles preventing the implementation of a national newborn screening programme in India are the lack of awareness among the stakeholders [9], insufficient budget allocation for health care, lack of government policies and the lack of political will. The health policies in India have typically targeted mortality and infectious morbidities but not disabilities. Inadequate health education, early hospital discharge and high number of out-of-hospital births are other reasons behind why neonatal screening has not been an important consideration for parents in India. Additionally, the absence of reliable and statistically relevant incidence data is a major limitation [10,11]. Despite all these obstacles, it is worth noting here that the government and policymakers in India have started recognizing the importance of newborn screening, which resulted in the implementation of the programme in the state of Kerala, Goa and Chandigarh [12]. In the year 2018, the Indian Council of Medical Research (ICMR) task force has recommended newborn screening of congenital hypothyroidism (CH) and congenital adrenal hyperplasia (CAH), considering the fact that CH is the commonest cause of preventable intellectual disability and CAH contributes significantly to both morbidity and early death [13]. The Indian Society for Pediatric and Adolescent Endocrinology has also published clinical practice guidelines for newborn screening, diagnosis, and the management of CH [14,15]. There were significant regional differences noted in the prevalence of hypothyroidism as per the ICMR study. For a country such as India with diverse ethnic groups and diverse dietary habits, it is essential to implement region-wise pilot newborn screening programme to identify the regional prevalence of IEM (inborn errors of metabolism) disorders.

We implemented a pilot NBS programme for the Udupi district of South India, with funding support from Grand Challenges Canada (GCC), the Government of Canada and Vision Group of Science and Technology (VGST), Government of Karnataka. The aim of the project was to study the need and viability for a newborn screening programme in India. The objectives were:

- To set up a newborn screening lab for the district.
- To assess the awareness of parents and community health care workers on NBS and IEM.
- To determine the incidence of congenital hypothyroidism (CH), congenital adrenal hyperplasia (CAH), glucose-6-phosphate-dehydrogenase deficiency (G-6PDD), galactosemia (GALT), biotinidase deficiency (BTD), and phenylketonuria (PKU) in the coastal district (Udupi) of South India.
- To analyze the benefit to cost ratio of having a newborn screening in place for prevalent IEM disorders in India.

## 2. Materials and Methods

A dedicated newborn screening lab was set up at the Centre of Excellence in Inborn Errors of Metabolism (CEIEM) at Manipal Academy of Higher Education, Manipal. Facilities of the lab include a dried blood spot puncher (Panthera-Puncher 9, Perkin Elmer, Turku, Finland), fluorescence reader (Victor 2D Fluorimeter, Perkin Elmer) with MultiCalc data processing software, plate washer (DELFIA washer-disk remover instrument, Perkin Elmer), shaker (DELFIA plate shaker, Perkin Elmer), incubator (TriNEST Incubator Shaker, Perkin Elmer), and −80 °C freezer (Sanyo Ultra Low Freezer, Shanghai, China). Whatman 903 filter paper for sample collection was purchased from Perkin Elmer and the demographic card was customized in house. Assay kits were purchased from Perkin Elmer. Brochure information sheets, leaflets and consent forms were developed both in the local language (Kannada) and in English. The information brochure was validated by a questionnaire-based survey among the parents visiting the obstetrics department of Kasturba Hospital, Manipal, and was revised accordingly. Reference ranges were fixed based on available literature on the Indian population and a reporting format was finalized [14,16,17].

Awareness-creation activities were adopted for parents and community health care workers (Anganwadi workers). One-to-one counselling was provided to parents with the help of a validated information brochure. Awareness-creation activities for Anganwadi workers were conducted using PowerPoint presentation sessions and street plays. To assess the awareness, a pre and post structured questionnaire was administered, consisting of socio-demographic, awareness, and attitude questions on NBS and IEM.

The programme was implemented in two phases. In the first phase, it was offered to the private hospitals in Udupi district namely Kasturba Hospital, Manipal, a tertiary care hospital and at two of its peripheral hospitals (Dr TMA Pai Rotary Hospital, Karkala and TMA Pai Hospital, Udupi). The screening was done for CH, CAH, G-6-PDD, PKU, GALT, and BTD for a period of 1 year from August 2017 to November 2018. Hands-on training was provided to nurses on counselling the parents, obtaining informed consent, heel-prick sample collection, filling demographic card and on transport and the storage of the sample. Parents were sensitized on the importance of NBS and were given information brochures during their antenatal visits at the Department of obstetrics, Kasturba Hospital, Manipal. A sample collection room was identified near the post-natal ward, equipped with a baby warmer and other required facilities and consumables. After counselling the parents on NBS and obtaining consent, those parents interested in participating in the programme were asked to bring their newborn child to the sample collection room after 48 h of birth and before 72 h. Using the special neonatal lancet, a heel prick was made, and samples were collected on the filter paper card. One drop of blood was filled in each circle (1/2 in. dia.) of the filter paper and collected up to a maximum of six circles per baby. These were then air-dried and maintained in a horizontal position for 2 h without touching each other. These samples were transported to the newborn screening lab on the same day and stored at −80 °C until analysis. Repeat sampling after 2 weeks was requested in the case of premature babies. Weekly and monthly updates were made on information such as total delivery, number of missed samples, reason for not collecting blood samples and for poor quality samples. The samples were brought to the newborn screening lab on the same day. The quality of the blood circles was checked as per the predetermined quality criteria and those failing to meet such criteria were rejected. Repeat sampling was requested through social workers in these cases. Screening was performed using the recommended tests and confirmatory tests were performed for the positive cases. Details of tests used for screening and confirmation are provided in Table 1. Positive cases obtained in the screening were reported to the treating doctor telephonically, by email and by WhatsApp. The social workers ensured that the affected child was called back for resampling for confirmatory tests.

Institutional ethical committee clearance was obtained for the study from the Manipal University Ethical Committee (MUEC). Informed consent form and patient information sheet (PIS) were developed in both the local language and in English and were approved by the institutional review board. All the parents were clearly informed about the procedure, risks and the benefits of the programme using PIS and information leaflets. Parents were never forced and were given enough time to make a decision on whether to participate in the screening programme.

In the second phase, the programme was extended to the government hospitals for sustainability. The phase 2 programme was implemented as directed by the Deputy Commissioner of Udupi district. The disorders to be screened for were decided at the district health meet based on the incidence data generated in the phase 1 programme. NBS for CH and CAH was implemented for a period of 1 year from April 2019 to March 2020 at all Government Maternity Hospitals in Udupi (Taluk general Hospital, Kundapura Taluk, Government Hospital, Karkala Taluk, and at the Maternity Hospital, Udupi) with funding from the Rashtriya Bal Swasthya Karyakram (RBSK) scheme of National Health Mission (NHM), Government of India. Consent forms were not collected in the second phase since it was implemented for all newborns as a government programme.

A cost benefit analysis of the newborn screening programme was performed by quantifying the disease burden in terms of disability-adjusted life years (DALY). Since true prevalence data on the IEM conditions across India are not available for this calculation, a pooled incidence was deduced from all the available reports across India.

## 3. Results

### 3.1. Awareness of IEM and NBS among Parents and Community Healthcare Workers

Results of the questionnaire-based survey on awareness among parents and Anganwadi workers of Udupi district on NBS and IEM are presented in the Supplementary Materials (Table S1). The age group of parents enrolled in the awareness survey ranged from 21 to 55 years, with a mean age group of 28.8 (±5.5). Among the total participants, 94.4% were females and 5.6% were males. More than half of them had an education level up to graduation (53.1%). Most of the participants were homemakers (46.9%). The awareness level among the participants on NBS and IEM was only 30% at the start of the study. However, the survey conducted after awareness-creation activities were conducted showed a significant increase to 98%. Around 96.3% of the parents were able to say that NBS diagnoses IEM before the symptoms appear. The majority of the participants (87.5%) declared that IEM are inherited and are present from birth. Additionally, 78.1% of the parents acknowledged that NBS is not a final confirmatory test for IEM. The entire study population agreed to the inclusion of NBS as a routine programme such as the immunization programme of the Indian Government. The majority (99.4%) of them agreed that they will recommend NBS for IEM to family members and relatives who are expecting babies.

### 3.2. Incidence Report

Health services in India are offered by both private and government sectors. As per the data published by the Statista Research Department in May 2021, there are 25,778 government hospitals and 43,486 private hospitals in India (nearly 1:2 ratio). Patients from the low-income group largely depend on the government hospitals, where all services are free, while those from the middle-income group prefer private hospitals. In order to understand the feasibility and sustainability of the programme in both private and government sectors, we implemented the programme in private hospitals in the first phase and extended to government hospitals in the second phase. A report of the incidence observed for both the phases of the programme during the study is presented in Table 2.

In the first phase, we collected 3913 samples and could screen 3514 (89.8%) babies from the 4380 deliveries in private hospitals for a period of 16 months, starting from August 2017 to November 2018. The number of samples rejected due to improper sampling/insufficient sample quantity was 44. There were 311 instances of repeat sampling mainly due to sample insufficiency, and to confirm the positive/borderline cases. In the second phase, screening was performed only for two disorders (CH and CAH) in the government sector. We collected 4700 samples and screened 4678 (97.8%) from a total of 4748 deliveries. Only 46 instances of resampling were performed in the second phase. The number of samples rejected due to insufficient sample quantity was 20.

Congenital hypothyroidism was confirmed by testing T4 (thyroxine) and TSH levels. Primary hypothyroidism was confirmed by the decreased levels of T4 with increased TSH levels. Treatment by administration of levothyroxine was started immediately for these cases and scanning was done to determine the etiological cause. In case of increased TSH with normal T4, a repeat TSH test was done after ruling out thyroid binding globulin deficiency. Treatment was started if TSH level was still high, and the baby was revaluated after 3 years to rule out transient hypothyroidism. From the 8113 babies screened for CH, 54 babies tested positive, and they were contacted for retesting and confirmation. Among them, 49 babies showed up for the retest and 10 babies were confirmed CH positive. The false positivity rate was 0.48%.

Congenital adrenal hyperplasia was confirmed by measuring serum 17-OHP, steroid and electrolyte levels. In confirmed cases, treatment was initiated with the administration of glucocorticoids. Genital reconstructive surgery was recommended in required cases. From the 8035 babies screened for CAH, 61 babies tested positive, and they were contacted for retesting. Among them, 55 babies showed up for the retest and three babies were confirmed as CAH positive. The false positivity rate was 0.65%.

Furthermore, G6PD deficiency was confirmed by quantitative whole blood G6PD assay. As G6PDD is an X-linked genetic condition, the severity of the deficiency in females varies based on the two alleles being heterozygous or homozygous. Genetic analysis was performed for the babies where possible. The baby is considered of severe condition if the G6PD activity in the confirmatory test is <30%. All the positive cases were treated for anemia and hyperbilirubinemia if present. They were counselled for the hemolytic risk triggered by various conditions such as acute infection, ingestion of fava beans and exposure to oxidative drugs such as antimalarials, etc. From the 2796 babies screened for CAH, 14 babies tested positive, and they were contacted for retesting and confirmation. Among them, 13 babies showed up for the retest and three babies were confirmed G6PDD positive. The false positivity rate was 0.36%.

Biotinidase deficiency was confirmed using a serum biotinidase enzyme assay. Cases with an enzyme activity <10% were considered as profound BTD and treatment with biotin administration was started immediately. A tandem mass analysis of blood for 3-hydroxy isovaleryl carnitine level, GC/MS of urine for 3-hydroxy isovaleric acid and genetic studies were also carried out for confirmation. From the 2949 babies screened for BTD, 29 babies were tested positive, and they were contacted for retesting. Among them, 22 babies showed up for the retest and two babies were confirmed BTD positive. Hence, the false positivity rate was 0.68%.

Galactosemia was confirmed by a repeat total galactose assay followed by Galactose-1-phosphate uridyltranferase enzyme assay and genetic studies. Treatment with a low-galactose diet was initiated immediately after confirmation. Parents were counselled to avoid lactose and galactose containing food items such as dairy products. From the 2680 babies screened for GALT, 18 babies tested positive, and they were contacted for retesting. Among them, 17 babies showed up for the retest and two babies were confirmed GALT positive. The false positivity rate was 0.56%.

A high fraction of the babies with positive screening results were not included in the follow up as they failed to show up for follow-up visits. Although the majority of deliveries happen in hospitals in urban area, many parents are from distant rural areas. Additionally, grandparents and other relatives play a key role in decision making related to newborns and they rely mainly on folk medicines and other home remedies.

### 3.3. Cost Benefit Analysis for Implementing a National Universal Newborn Screening Programme for CH, CAH and G-6PDD in India

True prevalence data on the IEM conditions across India are not available since no universal screening programme is present in the country. However, based on the incidence data in our study and other published data, we calculated a cost benefit estimate for universal newborn screening in India. For this purpose, pooled incidence data from the available reports are presented in Table 3.

A simplified cost for implementing the programme was computed from our phase 1 pilot programme, including the cost for follow-up testing, hospital visits and treatment (see Table 4).

We estimate that, the screening of any one disorder (for example CH in this instance) costs 6.45 USD. This cost is calculated from the recurring cost of 44,300 USD incurred (labor, assay kits, consumables, sample transport cost, confirmatory test cost and overhead charges) for screening 3869 samples for the six disorders in the first phase of our study. We estimate that the cost of screening an additional disorder is 1.5 USD. The cost incurred in our study matches the cost of screening in India reported by earlier studies [12]. Accordingly, considering the average number of births per day in India (67,385 as per UNICEF data [42]), a total cost of 434,633.25 USD (67,385 × 6.45 USD) is estimated to perform universal newborn screening for CH daily in India. The estimated cost of screening an additional condition (e.g., CAH, G-6PDD, etc.) would be 101,077.50 USD (67,385 × 1.5 USD).

The benefit was calculated by multiplying disability-adjusted life-year (DALY) with gross domestic product (GDP) per capita. DALY is the sum of years of lives lost (YLL) and years of life lived with disability (YLD). Since CH does not result in death, YLL is 0. YLD is the product of number of incident cases (I), disability weight (DW) and average duration of disability (years) (L). DW of 0.293 was taken as per WHO’s Global Health Estimates (GHE) for moderate Intellectual disability/mental retardation [43]. L was taken as 70, with the assumption that the disability would be manifested within 1 year of birth and that average life expectancy in India is 70 years [44].

In the case of G6PDD, YLL was calculated as the product of number of deaths (N) and standard life expectancy at age of death (in years) (L). As per the Institute for Health Metrics and Evaluation (IHME), 13,000 deaths were reported in 8.96 million cases globally [45]. This means that there would be one death in 690 cases of G6PDD. The incidence of G6PDD in India based on the pooled results from multiple previous studies was 1 in 118. Hence, out of the 67,385 babies born in a day in India, 571 babies are born with G6PDD. Without early detection and intervention, at least 1 death may occur. Hyperbilirubinemia is the common symptom seen in G6PD, and if left untreated, can cause severe motor and cognitive impairments. Accordingly, a DW of 0.542 was used in the calculation of YLD [43].

For CAH, the pooled incidence in India obtained from published reports was 1 in 4591. Hence, 15 babies are born a day with CAH. The number of deaths due to CAH has been investigated to a lesser extent, especially in India and other developing countries. Chatterjee et al. (1992) reported three deaths (37.5%) among eight CAH patients (7 females and 1 male) in a 2-year study conducted in Kolkatta, India [46]. A recent Indian study published in 2018 reported nine deaths (22.5%) out of forty CAH patients (32 females and 8 males) [47]. These two studies consisted of more females than males. This high female: male ratio severely underestimates the death rate since there is a chance that the affected males might have been undiagnosed or died undetected. However, owing to unavailability of the true mortality rate, an average death rate of 30% in CAH patients was assumed based on the above study. Accordingly, at least five deaths may occur if those 15 babies were not detected and treated for CAH. The fatal clinical manifestation of CAH is adrenal crisis, which can lead to death or morbidities such as mental retardation, or permanent neurological failure. Patients can also be presented with obvious physical deformity that makes others uncomfortable, which causes the person to avoid social contact, feel worried, sleep deprived, and think about suicide. [48,49,50,51]. For the calculation of YLD, DW of 0.128 and 0.405 was taken as per WHO’s Global Health Estimates (GHE) for mild intellectual disability/mental retardation and disfigurement, level 3, respectively [43].

Cost–benefit data are presented in Table 5.

## 4. Discussion

Implementation of a universal newborn screening programme in India is a topic of discussion that merits high importance. Many earlier workers in this area have identified the lack of awareness among the stakeholders as a major obstacle in implementing NBS in India. One of the key findings from our study is that the parents and Anganwadi workers (community health worker) were not very aware of IEM conditions and NBS prior to the awareness-creation activities we conducted. Parents were initially reluctant in providing consent for their newborns to participate in the study since they were unaware of IEM and the benefits of NBS. However, it was evident from the results of the post-interventional questionnaire-based survey conducted after the awareness programme that the majority of the parents and Anganwadi workers were convinced of the need for implementing a newborn screening programme in India. Anganwadi workers can play a key role in the successful implementation of a newborn screening programme in India because of their outreach.

Similar to our survey, Agarwal et al. studied the effectiveness of awareness creation activities using a questionnaire-based survey among the underprivileged population of Lucknow, India. They observed that 100% of the studied population was able to understand the advantages and methods of NBS after the awareness programme [53]. A survey in Bangalore, India showed that 79% of obstetricians, 62% of pediatricians, 95% of nursing staff and 99% of general public were not aware of newborn screening [9]. The challenges faced when initiating a newborn screening programme in rural area of Andhra Pradesh, India, owing to illiteracy, ignorance, taboos, superstitions and misbeliefs among the general public and a lack of awareness and interest among the medical community has been reported by Rama Devi et al. Regardless of the challenges, the programme was a success after repeated awareness programmes and trainings [54]. It is worth mentioning here the efforts taken by “The Indian Society for Pediatric and Adolescent Endocrinology” in distributing the information booklet on CH and CAH in English and Hindi for patients and health care workers. Similarly, RBSK provide resource materials and training to community healthcare workers for the identification of birth defects, deficiencies, developmental delays, and disabilities.

Our study to determine the incidence of IEM conditions in Udupi revealed an overall incidence of 16 IEM cases in the first phase of the programme after screening 3514 babies for six disorders. This means that there is an incidence of 1:220 for one or the other screened disorder in the population of Udupi district. Based on the incidence report obtained in the first phase of the study, Kasturba Hospital, Manipal, the major private hospital catering to the health services of the district decided to continue the screening programme at a cost to the parents. The incidence data was convincing for the district administration to implement the programme as a trial basis for two of the most prevalent conditions (CH and CAH) in the government maternity hospitals at no cost to the patient. The money allocation for the programme in the government was from the RBSK scheme of National Health Mission. The authors stress here the need for such studies across India to reveal the prevalence of IEM disorders so that health service providers and administrators are aware of the magnitude of the problem. It is worth mentioning here that the pediatricians and the administrators had no second thought in implementing the screening programme in the second phase after realizing the incidence rate.

Studies to establish the true incidence of IEM conditions in India is very limited. There were only 27 studies available to date that report the incidence of the selected IEM conditions in India, which are summarized in Table 3. From the pooled incidence data, a rough estimate of prevalence of the disorders in subjects under 10 years of age over a period of last 10 years considering zero mortality is deduced, of 277,400, 2,084,150 and 54,750 people with CH, G6PDD and CAH, respectively. All of the affected babies could lead a better-quality life if such disorders were detected and treated at an early stage from infancy. Another way of determining the prevalence and mortality rate of disorders would be to review hospital registries, which warrants a separate study. Even then, that data would not be entirely accurate, as affected children may not be brought to the hospital due to ignorance, with families instead resorting to folk medicine, or being deterred by the possibility of social stigma in cases of precocious puberty.

Considering the very high incidence rate and availability of effective treatment/management, we conducted a basic cost–benefit analysis (CBA) for implementing newborn screening for CH, G6PDD and CAH in India. The relative benefits of screening were found to be 6.91, 296.07 and 17.35 times for CH, G-6PDD and CAH, respectively, compared to the cost involved for the programme. It is to be noted that levothyroxine (for CH treatment), hydrocortisone and fludrocortisone (for CAH) is available in India at an affordable cost. This is a fortunate situation for India compared to so many other people living with CAH in other countries around the world.

As NBS is not established in India, the true prevalence data for these conditions is not available. The mortality rate for G-6PDD and CAH in India is also not available. For CBA calculation in this paper, mortality due to G6PDD was determined from the global data and mortality due to CAH was determined based on the two available Indian studies with a small cohort of affected patients with a skewed sex ratio. We would like to reiterate here that our CBA data for G-6PDD and CAH are underestimated and the actual benefit would be much higher if the actual mortality data were available for both these disorders.

A cost benefit analysis of newborn screening in India has not yet been reported as far as we are aware. The only such study was that of Agarwal et al., which reported a rough estimate of cost effectiveness for implementing universal NBS for CH in India, based on lost economic productivity due to undiagnosed CH cases [53]. All the reported CBAs on these disorders worldwide show similar results and have implemented the programme successfully for their babies. Yearly benefit to cost ratio calculated using DALY methodology for CH screening programme in Sri Lanka for the year 2011–2019 is reported to be in the range of 2.54–3.6 [55]. The benefit to cost ratio of CH newborn screening programme in the Philippines is reported to be 2.4 [56]. A study conducted in the US estimated a benefit to cost ratio for G-6PDD screening ranging from 1.38–3.42 using quality-adjusted life year (QALY) methodology [57]. The reported studies on the cost effectiveness of newborn screening for CAH is conducted by comparing the cost in screened and unscreened CAH patients [58,59,60,61]. The benefit to cost ratio of newborn screening in India that we have calculated is much higher than the already reported studies of other countries, confirming that the implementation of newborn screening in India will not be an economic burden but raise the standard of health quality.

The recent ICMR study in 2018 also proposed that it is time to address issues beyond survival, citing the fact that both neonatal and infant mortality rates have declined in some well performing states in India. The study reported a significant regional difference in the prevalence of CH and CAH and identified the challenges associated with implementing a universal newborn screening programme in urban and rural India. They recommended that newborn screening should be provided as a legal right of every newborn in the country [13]. The Indian Society for Pediatric and Adolescent Endocrinology has recommended that NBS for CH should be performed for every newborn in India, including preterm and low birth weight infants. They recommended TSH assay at 48–72 h of birth as the preferred screening test [14,15]. They also recommended the follow up and treatment regimens for CH.

It is worth mentioning here that Therrell et al. have stated in their paper that CH is the most cost-effective screened condition in most countries. They also mentioned in their review that screening of G6PDD is important for India [62]. Jalan et al. recommended that India should at least initiate screening of CH, CAH and G6PDD, which are common disorders in India [63]. Büyükgebiz considers NBS for CH as one of the major achievements in preventive medicine as neonates born with this disorder have a normal appearance and no detectable physical signs [64]. Tiwari et al., in their report on a pilot NBS study, stressed that India should initiate screening for CH, CAH and G6PD at a nominal cost [65]. It is also reported that hospitalization for CAH patients was reduced because of screening [58,59,66]. All these reports corroborate the findings from our study and proves that newborn screening for CH, G-6PDD and CAH can be implemented cost effectively in India and that the benefits of the programme far outweighs the cost.

## 5. Conclusions

Our study demonstrates that a properly designed awareness programme directed towards its stakeholders is important in expediting the implementation of a newborn screening programme in India.

The number of positive cases detected in our pilot programme points to the high prevalence of CH, CAH, G6PDD, BTD and GALT in the district of Udupi.

A cost benefit analysis showed that newborn screening can be implemented cost effectively in India. The CBA for CH, CAH, and G6PDD illustrates that the benefit of screening is higher than the cost of screening and treatment. The probable intellectual disability in 27,740 children yearly could be prevented in India with a universal newborn screening programme in place for CH. Similarly, it would help to prevent the possibility of life-threatening complications from hemolysis due to G-6PDD in 208,415 patients every year. Yearly, 5475 children in India could benefit from CAH screening via a reduction in morbidity, mortality and hospitalization associated with adrenal crisis, decline in virilization, normalized growth and puberty, and improved psychosocial environment. The authors reiterate that the data presented here is an extrapolation from the available reports and true prevalence data of these disorders are not available as of yet.

The study has clearly established the urgent need for a newborn screening programme in India and has demonstrated the viability of the programme for the most prevalent disorders. We strongly urge administrators in local and national health authorities to take the necessary steps to implement a viable, national-level newborn screening programme in the very near future.

## Figures and Tables

**Table 1:** Details of tests for screening and confirmation of the disorder.

Disorder	Screening Test	Confirmatory Tests
CH	hTSH: Time resolved fluoroimmunoassay (ELISA)	Repeat T4 and TSH on a fresh blood sample Optional-Thyroid scan (Radio nucleid) Genetic studies
CAH	17α-hydroxyprogesterone:Time resolved fluoroimmunoassay (ELISA)	Serum 17 OHP and Sr. Electrolytes ACTH stimulation test Steroid profiling Genetic studies
G6PDD	Fluorimetric assay (G6PD reduces NADP to NADPH when it oxidizes the substrate glucose-6-phosphate to 6-phosphogluconate)	Whole blood G6PD assay (Quantitative or qualitative) Genetic studies
BTD	Fluorimetric assay (Conversion of biotin 6-aminoquinoline(BAQ) to fluorescent 6-aminoquinoline (6-AQ) by biotinidase enzyme)	Serum biotinidase enzyme assay TMS of blood for C5-OH elevation and GC/MS of urine for 3-hydroxy isovaleric acid Genetic studies
GALT (Total Galactose)	Fluorimetry (Galactose oxidase method)	Repeat total galactose and galactose-1-phosphate assay GALT enzyme assay Genetic studies
PKU	Fluorimetry (Neonatal phenylalanine-fluorescent ninhydrin method)	Plasma aminoacids RBC DHPR Pterin analysis Genetic studies

**Table 2:** Report of number of cases of IEM conditions observed in Udupi district of South India.

Disorder Screened	Biomarker (Normal Levels)	Cut off Value Used	Number of Cases ^1,2 (*n*)^			Incidence Rate
			Phase 1	Phase 2	Total Incidence	
CH	TSH (<9 μU/mL)	>20 μU/mL	7	3	10/8113	1:811
CAH	17-α-OH-progesterone (<30 nmol/L)	>90 nmol/L)	2	1	3/8035	1:2009
G-6PDD	Glucose-6-phosphate dehydrogenase (>2 U/gHb)	<2 U/g Hb	3	Not done	3/2796	1:932
BTD	Biotinidase enzyme activity (>50 Units)	<30 Units	2	Not done	2/2949	1:1475
GALT	Total galactose (galactose + galactose-1-phosphate) (<8 mg/dL)	>11 mg/dL	2	Not done	2/2680	1:1340
PKU	Phenylalanine (<3 mg/dL)	>3 mg/dL	NIL	Not done	NIL	-

^1^ Number of babies screened in Phase 1 (*n*) = 3514. ^2^ Number of babies screened in Phase 2 (*n*) = 4678. The “number of babies screened”, mentioned in the foot note represents the total number of babies screened for one or the other disorder.

**Table 3:** Incidence based on pooled results on IEM conditions from published reports across India.

Condition	Studies Reporting Incidence	Total Samples Screened	Positive Cases	* Incidence (Pan India)
CH	Kochupillai et al., 1986 [18]; Desai et al., 1994 [19]; Rama Devi et al., 2004 [20]; Sanghvi et al., 2008 [21]; Lodh et al., 2013 [22]; Kapil et al., 2014 [23]; Gopalakrishnan et al., 2014 [16]; Anand et al., 2015 [24]; ICMR Task force 2017 [25]; Chaudhary et al., 2018 [26]; Kommalur et al., 2019 [27]; Sudheer Moorkoth et al. 2021 [Incidence reported in this paper]	235,651	266	1:887
G-6PDD	Khan et al., 1964 [28]; Saha et al., 1971 [29]; Seth et al., 1971 [30]; Flatz et al., 1972 [31]; Ghosh et al., 1981 [32]; Verma et al., 1990 [33]; Rama Devi et al., 2004 [20]; Pao et al., 2005 [34]; Kaur et al., 2010 [17], Lodh et al., 2013 [22]; Mohanty 2014 [35]; Goyal et al., 2015 [36]; Asghar et al., 2017 [37]; Bhasin et al., 2017 [38]; Samtani et al., 2017 [39]; Saraswathy et al., 2017 [40]; Kommalur et al., 2019 [27]; Verma et al., 2020 [41]; Sudheer Moorkoth et al. 2021 [Incidence reported in this paper]	89,342	758	1:118
CAH	ICMR study 2018 [13]; Rama Devi et al., 2004 [20], Kaur et al., 2010 [17]; Kommalur et al., 2019 [27]; Sudheer Moorkoth et al. 2021 [Incidence reported in this paper]	169,880	37	1:4591
BTD	Rama Devi et al., 2004 [20]; Lodh et al., 2013 [22]; Sudheer Moorkoth et al. 2021 [Incidence reported in this paper]	21,775	41	1:531
GALT	Lodh et al., 2013 [22]; Rama Devi et al., 2004 [20]; Gopalakrishnan et al., 2014 [16]; Kommalur et al., 2019 [27]; Sudheer Moorkoth et al. 2021 [Incidence reported in this paper]	68,033	15	1:4236
PKU	Lodh et al., 2013 [22]; Kommalur et al., 2019 [27]	41,627	8	1:5203

* Incidence is calculated from the published reports available from limited studies across the country and will not reflect the true incidence.

**Table 4:** Cost in USD for screening one disorder for a baby as per our study.

Cost Parameter	Cost for Screening One Disorder (CH)
Assay kit cost	1.10 USD
Sample collection and transport	0.85 USD
Labour (salary for programme coordinator, lab technician and general duty worker)	1.50 USD
Lab consumables and chemicals	1.15 USD
Confirmatory Visits and treatment (Mean cost for laboratory test; productivity loss of accompanying person; transportation; professional fee; medication)	0.85 USD
Overhead	1 USD
Total screening cost per baby for one disorder	6.45 USD ^1^
With an extra cost of 1.5 USD/per baby, an additional disorder could be screened	

^1^ Exchange rate 1 USD = 75.13 INR. CH: congenital hypothyroidism.

**Table 5:** Cost–benefit analysis for screening 67,385 babies (average number of births per day in India) for CH, G6PDD and CAH per day.

Condition Screened	Years of Life Lost (YLL)	Years Lived with Disability (YLD)	DALY (YLL + YLD)	GDP per Capita * (USD)	Benefit (DALY × GDP) (USD)	Cost of Screening (USD)	Benefit: Cost Ratio
CH	0	1558.76	1558.76	1927.70	3,004,821.65	4,34,633.25	6.91
G-6PDD	50	15,474.10	15,524.10	1927.70	29,925,807.57	101,077.50 **	296.07
CAH	350	559.65	909.65	1927.70	1,753,532.31	101,077.50 **	17.35

* GDP per capita of India as per the World Bank 2020 data [52]; ** Additional cost for adding this disorder to an established NBS programme for CH.

## Data Availability

Original data on the incidence is provided as Supplementary Materials Table S1.

## Supplementary Materials

The following supporting information can be downloaded at: https://www.mdpi.com/article/10.3390/ijns8020026/s1. Table S1, Results of questionnaire-based survey on awareness among parents and Anganwadi workers of Udupi district on NBS and IEM.
