# Supplementary material for: Need and Viability of Newborn Screening Programme in India: Report from a Pilot Study
Source: Int J Neonatal Screen. 2022 Mar 29;8(2):26. doi: 10.3390/ijns8020026 (PMC9036214; doi:10.3390/ijns8020026)
Supplement: Supplementary file 1 [file IJNS-08-00026-s001.zip › IJNS-1557744-supplementary.pdf]

Table S1: Results of questionnaire based survey on awareness among parents and Anganwadi workers on NBS and IEM

	Questionnaire	Post intervention Response			
		**Parents[1(n)]**		**AWW[2(n)]**	
		Yes (%)	No	Yes (%)	No
Awareness questions	NBS helps to diagnose IEM before symptoms appear.	154(96.3)	6	385(94.8)	21
	IEM disorders are inherited and parent from birth.	140(87.5)	20	345(85)	61
	IEM is not infectious.	154(96.3)	6	303(74.6)	103
	Do you know that IEM are rare diseases?	142(88.8)	18	364(89.7)	42
	NBS should be done after 48 h and before 72 h of birth and before discharge of newborn baby from hospital.	122(76.3)	38	373(91.9)	33
	IEM should be diagnosed as early as possible to prevent death or disability.	76(47.5)	84	394(97.0)	12
	Infants with IEM (treatable) can lead a normal life if diagnosed and treated at early neonatal period.	107(66.9)	53	391(96.3)	15
	Do you agree that babies with IEM look healthy like other newborns when they are born?	110(68.8)	50	283(69.7)	123
	Do you know that NBS is done by collecting only 2-3 drops of blood from baby's heel?	157(98.1)	3	349(86)	57
	Do you know that there are no risks involved in performing NBS?	131(81.9)	29	378(93.1)	28
Attitude questions	Do you agree that newborn screening should be made a routine program like immunization program?	160(100)	0	366(90.1)	40
	Would you like to receive information on NBS prior to your babies birth?	152(95.0)	8	NA	NA
	Are you willing to be a part of NBS Program in the sample collection and in counselling parents?	NA	NA	367(90.4)	39
	Would you recommend NBS for any of your family members or neighbors who are expecting a baby?	159(99.4)	1	395(97.3)	11
	Would you like to receive training regarding NBS?	NA	NA	384(94.6)	22
	Do you think that the public awareness programs regarding screening test conducted by Government, health workers or other media is helpful in creating awareness among people?	142(88.8)	18	384(94.6)	22
